# Characterization of 3D-Printed Ti-6Al-4V Alloy Behavior During Cold Deformation

**DOI:** 10.3390/ma18081832

**Published:** 2025-04-16

**Authors:** Tin Brlić, Stoja Rešković, Sonja Kraljević Šimunković, Ljerka Slokar Benić, Samir Čimić

**Affiliations:** 1 Faculty of Metallurgy, University of Zagreb, Aleja Narodnih Heroja 3, 44000 Sisak, Croatiaslokar@simet.unizg.hr (L.S.B.); 2 School of Dental Medicine, University of Zagreb, Ivana Gundulića 5, 10000 Zagreb, Croatia; kraljevic@sfzg.hr

**Keywords:** additive manufacturing, 3D-printed Ti-6Al-4V alloy, cold deformation, thermography, digital image correlation (DIC)

## Abstract

In this paper, the characterization of the deformation behavior of additively manufactured 3D-printed Ti-6Al-4V alloys during elastic and plastic deformation was carried out on the test samples deformation zone during cold deformation at room temperature. The additive manufacturing process direct metal laser sintering (DMLS) was used to 3D print the Ti-6Al-4V test samples. The temperature, i.e., stress, changes, strain, and strain rate distribution in the deformation zone of the 3D-printed Ti-6Al-4V alloy during elastic and plastic deformation were compared using static tensile tests, thermography, and digital image correlation (DIC) simultaneously. Periodic oscillations of the maximum temperature changes during elastic and plastic deformation were observed in the deformation zone. The thermoelastic effect with the lowest temperature drop between −0.47 °C and −0.54 °C was observed in the deformation zone of the 3D-printed Ti-6Al-4V testing samples during elastic deformation. A significant difference between strain and strain rate localization in the deformation zone was found immediately before fracture of the test sample. Maximum strain amounts in the range of 0.078–0.080 and strain rates of 0.025–0.027 s^−1^ were determined. Static tensile tests, thermography, and digital image correlation were proved to be valid methods for determining the localization of stress, strain, and strain rate in the deformation zone of 3D-printed Ti-6Al-4V test samples.

## 1. Introduction

Titanium has a low density and is a lightweight material. It can be alloyed with various elements. Titanium alloys are widely used due to their optimal properties, such as mechanical strength, biocompatibility, corrosion resistance, etc. [1,2,3,4,5,6,7]. They have applications in the aerospace industry, automotive industry, and biomedicine, mostly for biomedical implants [8].

In biomedicine, the use of titanium alloys is considered desirable and successful, especially in the fields of dentistry and orthopedics. This is because resistance to various loads is required, especially under significant cyclic dynamic loads in orthopedics for products such as shoulders, hips, knees, ankles, etc., where titanium alloys have proven to be a very good material choice. At the same time, titanium alloys in dentistry have to meet certain mechanical load requirements due to their application in various dental products such as implants, dental posts, screws, abutments, braces, and instruments. The importance of titanium alloys in dentistry is also demonstrated by their application in manufacturing of brackets and wires. Titanium alloys have a good strength-to-weight ratio and are corrosion-resistant, which makes them desirable materials in dentistry [1].

A well-known and frequently used titanium alloy in the biomedical and aerospace industries is the alloy Ti-6Al-4V. The Ti-6Al-4V alloy is widely used in various fields due to its properties. It has a good combination of mechanical strength, corrosion resistance, and low weight [9]. In addition to excellent mechanical properties, its cost-effectiveness is also favorable. There are different types of Ti-6Al-4V alloys, the most common of which is the grade 5 alloy. In addition, there is a Ti-6Al-4V alloy Grade 23 ELI (Extra-Low Interstitials), which has a lower percentage of interstitial elements, which increases the ductility and fracture toughness of these alloys [1].

Titanium alloy products, including Ti-6Al-4V alloys, are largely manufactured using conventional production methods, mainly through various metal-forming technologies such as forging and rolling. Considering the material consumption, cost, and machining of alloys in this type of production, which is not easy, additive manufacturing is used to produce various complex Ti-6Al-4V products. In both traditional and additive manufacturing, knowledge of the deformation behavior of the 3D-printed Ti-6Al-4V alloy during metal forming is of crucial importance [9,10].

The deformation process of metallic materials is investigated using various methods. A literature review shows that digital image correlation (DIC) is frequently used to clarify the deformation behavior or strain localization [11,12]. In addition to digital image correlation, thermography is also often used to investigate the deformation behavior of metallic materials and alloys [13,14,15]. These methods are used for the investigation of various metallic materials such as mild steel [16], micro-alloyed steel [17], stainless steel [13], aluminum–magnesium alloys [18], nickel alloys [19], and titanium alloys [20]. Thermography and digital image correlation are frequently used to determine temperature and strain localization, i.e., the homogeneous and inhomogeneous deformations of the metallic materials plastic flow. The determination of the maximum temperature change distribution, which under certain conditions can be associated with stress changes in the deformation zone of test samples, is of great importance when thermography and digital image correlation are used simultaneously. The application of these methods is often used during the deformation process of metallic materials, most often during static tensile tests [21].

Digital image correlation (DIC) has found application in the examination of the Ti-6Al-4V alloy deformation behavior and localization. In the study [22], digital image correlation was used to determine the strains of the conventional and 3D-printed Ti-6Al-4V alloy. DIC was also used in the research of the conventional bimodal Ti-6Al-4V alloy [23], where the distribution of strains was monitored during the deformation process. In another study of the strains in the deformation zone of the conventional Ti-6Al-4V alloy during the deformation process, DIC was also used [24]. The Ti-6Al-4V alloy produced by LENS technology was investigated by DIC during the strain localization [25]. The plastic deformation of a conventional Ti-6Al-4V alloy was determined by DIC in another study [26].

Since deformation processes occur during the production and application of Ti-6Al-4V alloys, studies have been carried out in this research area to obtain information about their mechanical properties and deformation behavior under different conditions [9,22,25,27,28,29,30]. There are various studies on the mechanical properties of Ti-6Al-4V alloys. In [22,27], the authors compared the mechanical properties of Ti-6Al-4V alloys produced by turning (drawn bars) and by additive manufacturing (direct metal laser sintering—DMLS) during a static tensile test. A comparison of the mechanical properties of a 3D-printed Ti-6Al-4V alloy produced by the selective laser melting (SLM) method was carried out at different temperatures during the tensile test [9]. The mechanical properties and fracture surface of the Ti-6Al-4V ELI Grade 23 alloy produced by the laser powder bed fusion (LPBF) process were also investigated during a static tensile test at room temperature [28].

Recently, more intensive research of the Ti-6Al-4V deformation behavior has been carried out [23,24,26,31,32,33,34]. The deformation behavior and plastic flow of the Ti-6Al-4V alloy obtained by conventional production (rolled from a thin sheet) were investigated [26] at different strain rates during tensile tests. The behavior of the conventional Ti-6Al-4V alloy during plastic deformation under static and dynamic loading was investigated in [31]. This study was carried out at high strain rates where the strains were localized in the deformation zone. The strain behavior of a conventionally produced Ti-6Al-4V alloy (by electrical discharge machining) was carried out in the study [24]. In the study [23], the tensile deformation behavior of a conventional bimodal Ti-6Al-4V alloy was studied at different deformation degrees. The plastic flow of a 3D-printed Ti-6Al-4V alloy produced by the SLM method and a conventional Ti-6Al-4V alloy was investigated in [32]. A high instability of the plastic flow of the 3D-printed Ti-6Al-4V alloy at high temperatures was found compared to the conventional Ti-6Al-4V alloy.

A literature review leads to the conclusion that studies on deformation behavior have been conducted more frequently on conventionally produced Ti-6Al-4V alloys, while studies on 3D-printed Ti-6Al-4V alloys produced by additive manufacturing are significantly rarer. In fact, there are only a limited number of studies on this topic, and the available information is not sufficient to explain the additively manufactured Ti-6Al-4V alloys deformation behavior. Therefore, this research is important due to new scientific knowledge about the localization and distribution of stress, strain, and strain rate during elastic and plastic deformation in the deformation zone. In order to gain new scientific knowledge in the field of stress, strain, and strain rate localization and distribution, this study will determine the possibility of using thermography and digital image correlation in static tensile tests on 3D-printed Ti-6Al-4V test samples during elastic and plastic deformation. Indeed, it is important to characterize the deformation behavior of the 3D-printed Ti-6Al-4V alloy as it is increasingly used in various fields such as biomedical implants, dentistry, and the aerospace industry. The obtained new scientific knowledge about the stress, strain, and strain rate localization and distribution will contribute to a better understanding of the risk of excessive and inhomogeneous distribution and localization in certain areas of the 3D-printed Ti-6Al-4V alloy products, which can lead to damage and premature cracking and fracture of the product.

The aim of this paper was to research the deformation behavior of a 3D-printed Ti-6Al-4V alloy during elastic and plastic cold deformation using simultaneous static tensile tests, thermography, and digital image correlation. The 3D-printed Ti-6Al-4V test samples were examined for the determination of temperature, i.e., stress changes, strain, and strain rate distribution and localization in the deformation zone.

## 2. Materials and Methods

### 2.1. Additively Manufactured Test Samples

**Figure 1:**
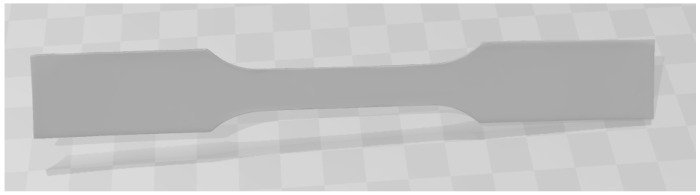
Ti-6Al-4V test sample in STL format.

The Ti-6Al-4V test samples were used for the investigation in this study. They were prepared as a CAD file (3D model), converted to standard tessellation language (STL) file format, and then sent to the 3D printer (Figure 1). A CAD program (Dassault Systems, Solidworks 2023 SP2.1) was used to obtain a 3D model (STL file) of the test sample for the printing process. The Materialize Magics 25.02 (Materialise HQ, Leuven, Belgium) slicer was used to prepare the STL model for 3D printing, i.e., for setting up the support, and EOSPRINT 2.13 for positioning the model with the support on the desktop and for selecting the relevant printing parameters.

The test samples for the static tensile tests during cold deformation were produced using the EOS M290 3D printer (EOS GmbH, Krailling, Germany) with the working platform dimensions of 250 mm × 250 mm × 325 mm. The EOS M290 3D printer used a Yb-ber laser (1 × 400 W) and the direct metal laser sintering (DMLS) method. The main parameters used during the printing process are listed according to Equation (1) in Table 1.

(1) $$Energy\;Input\left(\frac{J}{{mm}^{3}}\right)=\frac{Laser\;Power}{Laser\;Speed\times Layer\;Thickness\times Hatch\;Distance}$$

**Figure 2:**
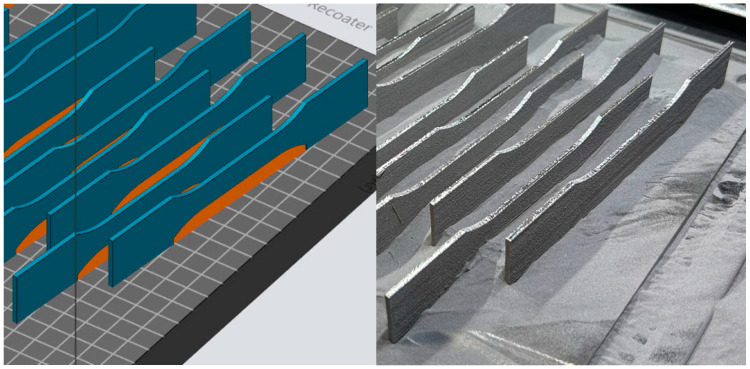
Printed direction of Ti-6Al-4V test samples.

The printing direction of the test samples was the longitudinal direction, which indicates the laser scanning direction (Figure 2).

EOS titanium Ti64 Grade 23 powder was used for the additive manufacturing of rectangular Ti-6Al-4V test samples. It is classified as a Grade 23 titanium alloy according to ASTM B348. The chemical composition complies with ASTM F136, ASTM F3001, and ASTM F3302 (Table 2). The metallic Ti64 Grade 23 powder has a particle size and distribution of 20–80 µm.

The titanium powder Ti64 Grade 23 was repeatedly laminated onto the solidified layer during the 3D printing process of Ti-6Al-4V alloy test samples. After the printing process, the test samples were cut from the working platform.

### 2.2. Static Tensile Test

**Figure 3:**
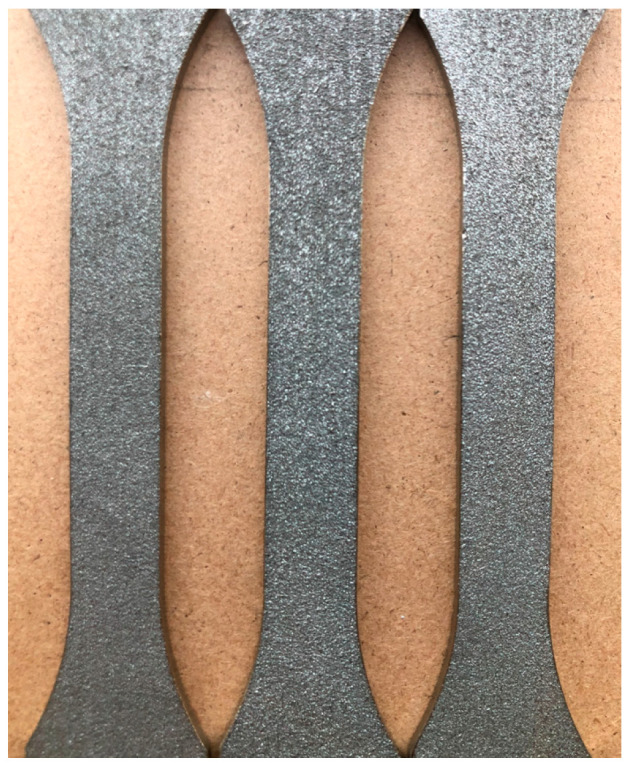
The 3D-printed Ti-6Al-4V test samples for tensile testing produced with DMLS technology.

The dimensions of the rectangular 3D-printed Ti-6Al-4V test samples were 40 mm in original gauge length, 10 mm in original width, and 2 mm in thickness (Figure 3). Static tensile tests, thermography, and digital image correlation (DIC) were used during the cold deformation of the test samples. The Hegewald & Peschke Inspect table 100 tensile testing machine was used for static tensile tests at a stretching rate of 10 mm/min.

Various test points during the cold deformation of the 3D-printed Ti-6Al-4V test samples were determined during the static tensile test.

### 2.3. Thermography

Thermography and digital image correlation tests were simultaneously used with the static tensile tests. The black matte coating Colormatic Lechsys 29141 RAL 9005 (Wolvega, The Netherlands) with an emissivity factor of 0.95 was used for the test sample preparation. The infrared camera VarioCAM^®^M82910 (JENOPTIK, Jena, Germany) with a temperature sensitivity of 80 mK was used to determine the temperature distribution during cold deformation. Furthermore, 50 frames per second were taken during the tests. The distance between the infrared camera and the sample was 0.59 m. The software package IRBIS3 professional (v.3.0.0.145) was used to analyze the temperature distribution of the deformation zone.

### 2.4. Digital Image Correlation (DIC)

**Figure 4:**
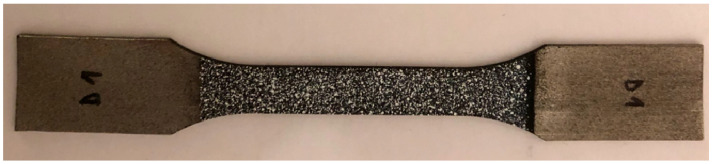
The 3D-printed Ti-6Al-4V test samples prepared for thermographic and digital image correlation tests.

The strain distribution of the test samples deformation zone during the static tensile tests was determined using the Basler ace acA2500-14um digital camera (Basler AG, Ahrensburg, Germany) with a resolution of 5 MPix. The camera was mounted perpendicular to the test sample. The distance between the digital camera and the sample was 0.19 m. The test samples were prepared by applying Colormatic Lechsys 29141 RAL 9010 white markers (Wolvega, The Netherlands) to the black matte surface (Figure 4). Five frames per second were taken during the tests. The Zeiss Quality Suite: Inspect Correlate 2023 software package was used to analyze the strain distribution of the test sample deformation zone.

**Figure 5:**
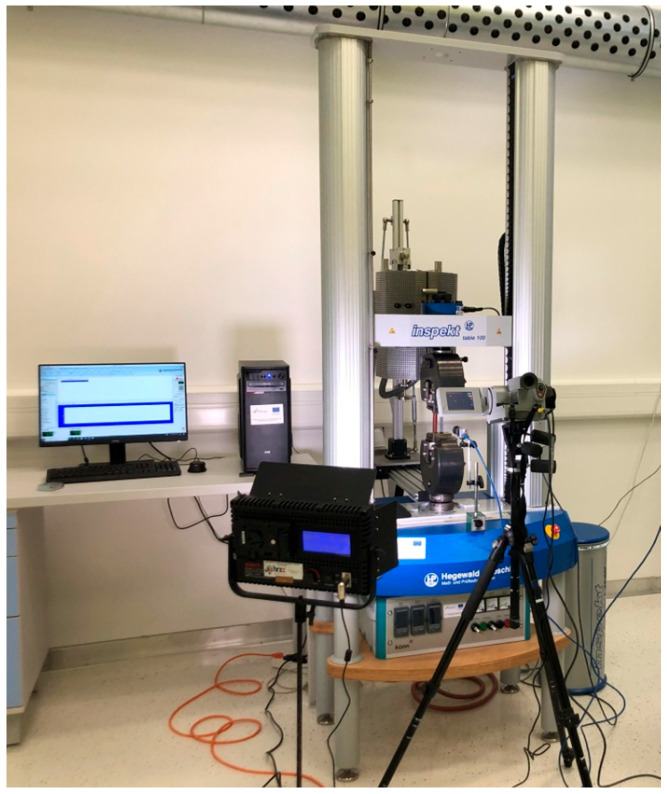
Equipment layout for the testing of 3D-printed Ti-6Al-4V samples.

The static tensile test, thermography, and DIC equipment layout for testing of samples are shown in Figure 5.

## 3. Results and Discussion

**Figure 6:**
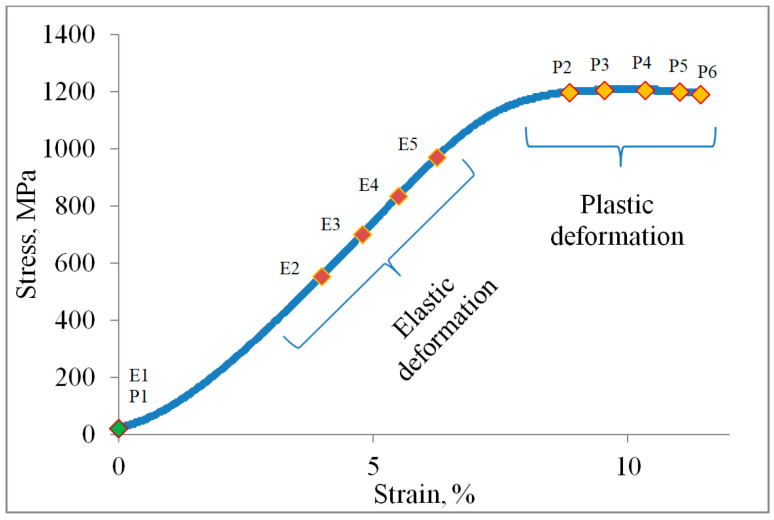
Stress–strain diagram of the 3D-printed Ti-6Al-4V alloy.

The results of temperature, i.e., stress, strain, and strain rate distribution of 3D-printed Ti-6Al-4V alloy, were investigated during the time evolution involving both elastic and plastic deformation. The stress–strain diagram in Figure 6 shows the points of elastic and plastic deformation that are the subject of the present research. Points E1–5 (elastic) and P1–6 (plastic) during the deformation process were selected for this study.

The homogeneous deformation of the 3D-printed Ti-6Al-4V alloy can be seen in Figure 6. The stress–strain diagram characterizes the homogeneous transition from the elastic to the plastic region of deformation without phenomena such as Lüders bands and the Portevin–Le Chatelier (PLC) effect, which are characteristic of some conventional alloys such as micro-alloyed steels, aluminum–magnesium, and titanium alloys.

**Figure 7:**
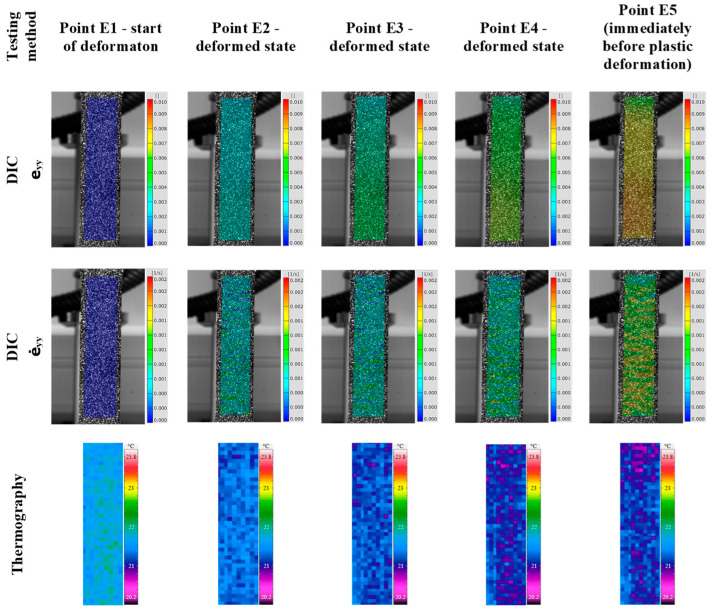
Qualitative analysis of 3D-printed Ti-6Al-4V alloy during elastic deformation.

**Figure 8:**
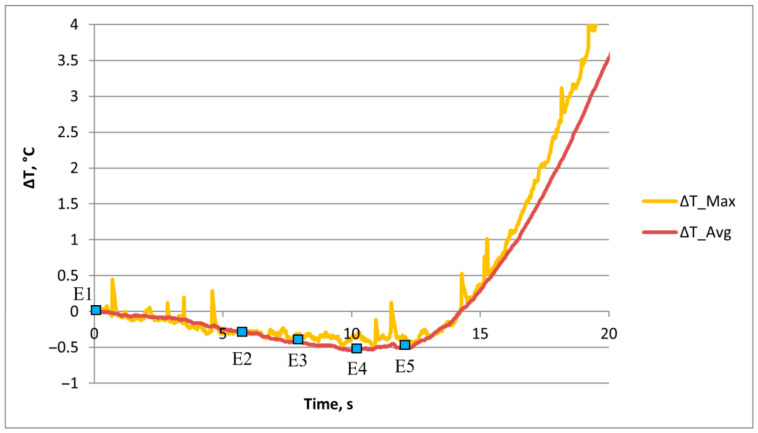
Temperature drops in 3D-printed Ti-6Al-4V alloy during elastic deformation (points E1–E5).

**Figure 9:**
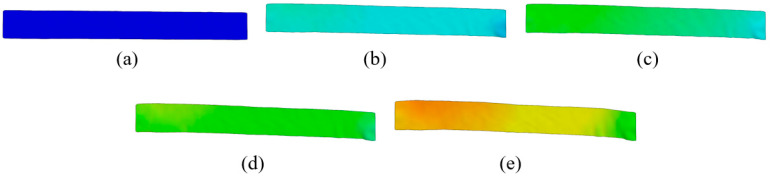
The 3D visualization of strain distribution during elastic deformation in (**a**) point E1, (**b**) point E2, (**c**) point E3, (**d**) point E4, and (**e**) point E5.

**Figure 10:**
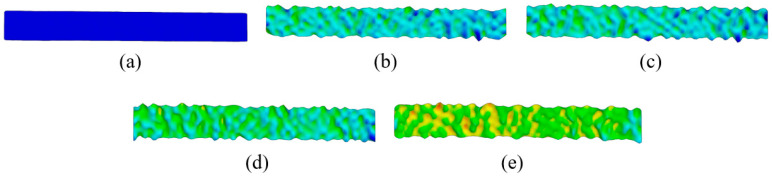
The 3D visualization of strain rate distribution during elastic deformation in (**a**) point E1, (**b**) point E2, (**c**) point E3, (**d**) point E4, and (**e**) point E5.

**Figure 11:**
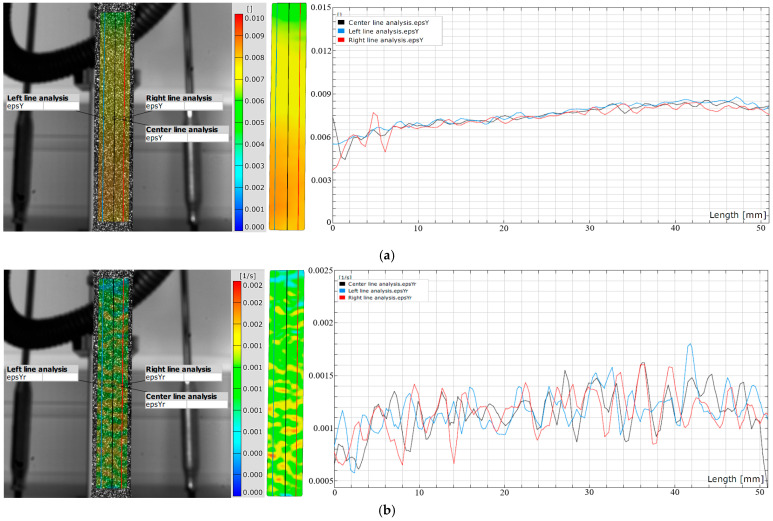
DIC line analysis area of (**a**) elastic strain point E5 and (**b**) elastic strain rate point E5.

**Figure 12:**
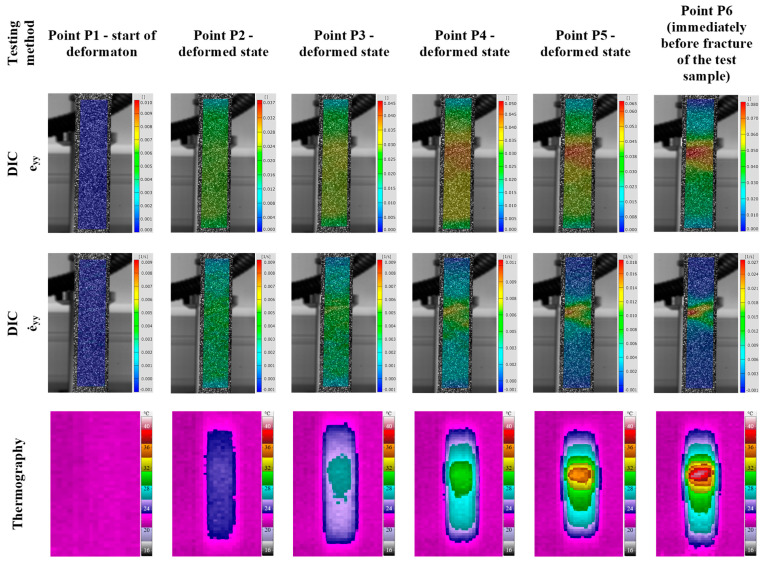
Qualitative analysis of 3D-printed Ti-6Al-4V alloy during plastic deformation.

**Figure 13:**
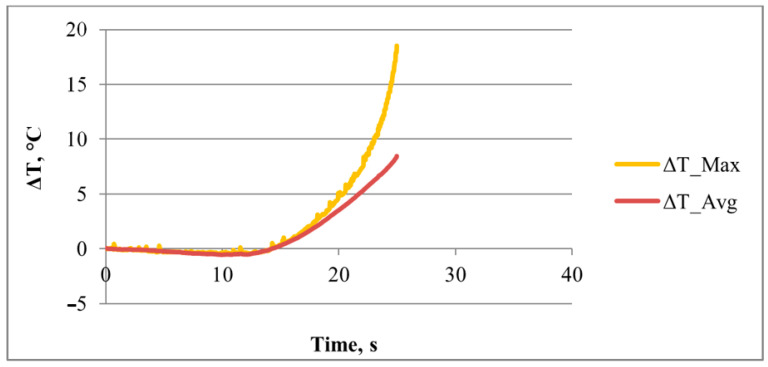
Diagram of time–temperature change of 3D-printed Ti-6Al-4V alloy during cold elastic and plastic deformation.

**Figure 14:**
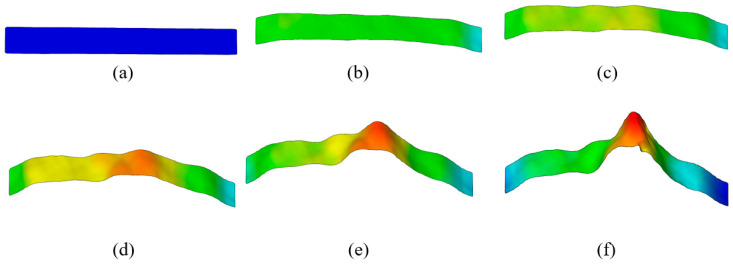
The 3D visualization of strain distribution during plastic deformation in (**a**) point P1, (**b**) point P2, (**c**) point P3, (**d**) point P4, (**e**) point P5, and (**f**) point P6.

**Figure 15:**
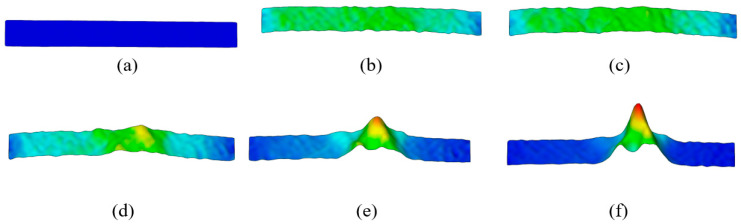
The 3D visualization of strain rate distribution during plastic deformation in (**a**) point P1, (**b**) point P2, (**c**) point P3, (**d**) point P4, (**e**) point P5, and (**f**) point P6.

**Figure 16:**
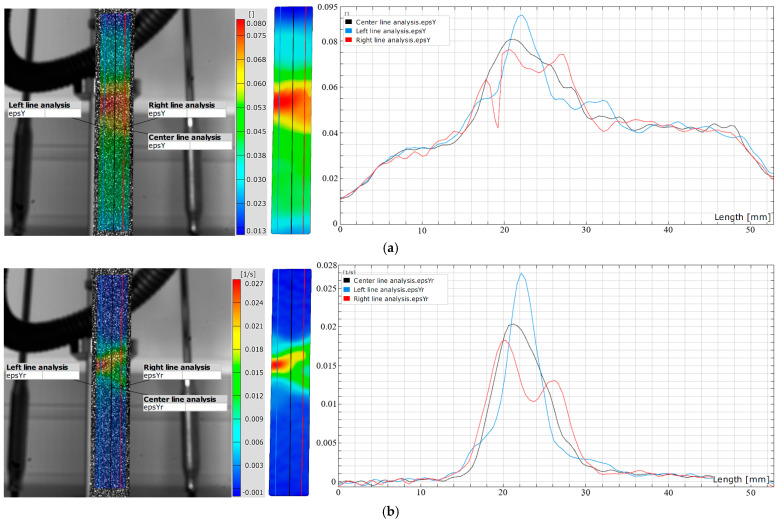
DIC line analysis area of (**a**) plastic strain point P6 and (**b**) plastic strain rate point P6.

Since the conventional method of static tensile testing cannot provide significant information about the local distribution of stresses, strains, and strain rates during elastic and plastic deformation, the qualitative and quantitative analysis of thermography and digital image correlation were used to study the deformation zone of 3D-printed Ti-6Al-4V test samples. Therefore, the qualitative and quantitative results of thermography and digital image correlation were obtained for the 3D-printed Ti-6Al-4V alloy (Figure 7, Figure 8, Figure 9, Figure 10, Figure 11, Figure 12, Figure 13, Figure 14, Figure 15 and Figure 16). The results of the maximum temperature, i.e., stress, change, strain (e_yy_), and strain rate (ė_yy_) distribution of the test samples were analyzed. The marked deformation zone of the test sample shows the region of interest for the temperature, i.e., stress, change, strain, and strain rate distribution (Figure 7 and Figure 12).

The analysis of the elastic and plastic deformation of the 3D-printed Ti-6Al-4V test samples was divided into different research points (Figure 6, Figure 7 and Figure 12). For the elastic deformation, points E1–5 and P1–6 for the plastic deformation were selected in this research. Points E1 and P1 refer to the start of deformation while points E2–E4 and P2–P6 relate to the different deformation degrees during elastic and, plastic cold deformation of the 3D-printed Ti-6Al-4V alloy. Point E5 refers to the moment immediately before the plastic deformation, while point P6 is immediately before the fracture of the test sample.

The qualitative results of temperature, i.e., stress, change, strain (e_yy_), and strain rate (ė_yy_) distribution of the 3D-printed Ti-6Al-4V alloy during elastic deformation are shown in Figure 7.

The qualitative results in Figure 7 show that clear determination of temperature, i.e., stress, changes, strain, and strain rate distribution, using thermography and digital image correlation in the 3D-printed Ti-6Al-4V sample during elastic deformation can be made. The results also show that in the area of elastic deformation, it is possible to determine even distribution of low temperature, i.e., stress, strain, and strain rate changes.

The temperature drop during elastic deformation of the 3D-printed Ti-6Al-4V alloy was determined from the coloration of the test sample deformation zone during elastic deformation (points E2–E5) (Figure 7). In conventionally produced metallic materials, such as steel alloys (low-carbon steel), this behavior and temperature drop during elastic deformation are associated with the phenomenon known as the thermoelastic effect [35]. Therefore, a more detailed quantitative analysis, using a thermography time diagram for maximum and average temperature changes during elastic deformation, was conducted (Figure 8).

The diagram of time–temperature change during elastic deformation clearly shows that a temperature drop, or negative temperature change (before the start of plastic deformation), occurs in the 3D-printed Ti-6Al-4V alloy, which is characteristic of the thermoelastic effect during the deformation process (Figure 8). The lowest temperature drop of −0.47 °C was measured for maximum temperature changes and −0.54 °C for the average temperature changes in the 3D-printed Ti-6Al-4V alloy.

The start of plastic flow was determined at the moment when the first temperature change increase occurred (Figure 8). The periodic oscillations of the maximum temperature changes were determined during the elastic deformation.

In addition to the analysis with thermography, a more detailed qualitative and quantitative analysis with digital image correlation was carried out. The detailed digital image correlation qualitative analysis was performed using DIC line analysis and the 3D view of the results to clearly visualize the distribution of strain and strain rate during elastic deformation (Figure 9, Figure 10 and Figure 11).

The analysis of strain and strain rate 3D visualization was performed at points E1–5 during elastic deformation (Figure 9 and Figure 10). DIC line analysis results are in detail determined in point E5 (maximum elastic deformation) to determine the maximum strain and strain rate distribution during elastic deformation (Figure 11). The line analysis was performed using three lines along the deformation zone of the test sample: left (blue), middle (black), and right (red) line (Figure 11).

The results clearly show that the strain distribution in the deformation zone of the 3D-printed Ti-6Al-4V alloy test sample was not uniformly distributed along the length of the test sample. It was determined that the strain amounts are slightly higher in one part of the test sample during elastic deformation (point E2–E5) (Figure 9 and Figure 11a).

At the same time, strain rate distribution was different relative to the strain distribution, as it changed randomly over the length of the test samples (Figure 10 and Figure 11b). Both strain and strain rate amounts increased with increasing degrees of elastic deformation (points E2–E5) (Figure 9 and Figure 10).

The quantitative results (Figure 11) show higher oscillations in strain rate amounts along the test sample related to the strain distribution during elastic deformation of the 3D-printed Ti-6Al-4V alloy at point E5. The same behavior was also determined for all other points, i.e., E2–E4. No significant localization of strain and strain rate was observed during elastic deformation in the deformation zone of the test samples. The determined deformation behavior during elastic deformation is related to the microstructure changes in the 3D-printed Ti-6Al-4V alloy. These changes were more intense and significant during plastic deformation.

Research into the deformation behavior in the area of plastic deformation of the 3D-printed Ti-6Al-4V alloy was carried out after the investigation of the deformation behavior in the elastic deformation region. The qualitative results of the temperature, i.e., stress, change, strain (e_yy_), and strain rate (ė_yy_) distribution of the 3D-printed Ti-6Al-4V alloy during plastic deformation are shown in Figure 12.

The results show that a different distribution and higher temperature, i.e., stress, change, strain, and strain rate amounts were determined during plastic deformation compared to the elastic deformation of the 3D-printed Ti-6Al-4V alloy (Figure 12). The temperature, i.e., stress, change, strain, and strain rate amounts increased with the increase in deformation degree (points P2–P6) during cold plastic deformation. As the plastic deformation increased, the localization of stress, strain, and strain rate was located in the central part of the 3D-printed Ti-6Al-4V test sample deformation zone (Figure 12).

A detailed analysis of temperature, i.e., stress, changes was performed using quantitative analysis of the thermography time diagram for the maximum and average temperature changes during plastic deformation (Figure 13).

The diagram of time–temperature change clearly shows the intensive temperature, i.e., stress, change increase after the start of plastic deformation. The maximum temperature, i.e., stress, changes are determined at point P6 immediately before fracture of the 3D-printed Ti-6Al-4V test sample (Figure 12 and Figure 13). The highest temperature values of 18.53 °C (for the maximum temperature changes) and 8.46 °C (for the average temperature changes) were measured for the entire deformation zone of the 3D-printed Ti-6Al-4V test sample during the deformation process. In Figure 13, the time–maximum temperature change was also determined for periodic oscillations during plastic deformation. These oscillations of maximum temperature, i.e., stress, changes during elastic and plastic deformation are related to the microstructure changes, and the exact cause of the oscillations needs to be further investigated.

A detailed qualitative and quantitative analysis of the plastic deformation with digital image correlation was performed using DIC line analysis and 3D visualization of the results (Figure 14, Figure 15 and Figure 16).

The 3D visualization analysis of strain and strain rate distribution was performed at points P1–6 during plastic deformation (Figure 14 and Figure 15). The results of DIC line analysis were obtained at point P6 (maximum localization of plastic deformation) to determine the strain and strain rate distribution (Figure 16). The areas of DIC line analysis are the same as for elastic deformation—the left (blue), middle (black), and right (red) line (Figure 16).

The results clearly show that the strain distribution in the deformation zone of the 3D-printed Ti-6Al-4V alloy test sample was not uniformly distributed along the length of the test sample (points P3–P6). The strain and strain rate localization were determined in the central part of the testing sample deformation zone (points P4–P6) (Figure 12, Figure 14 and Figure 15). Both strain and strain rate amounts were higher as the degree of plastic deformation increased (points P2–P6) (Figure 14 and Figure 15).

This localization was in the central part of the test sample deformation zone (points P4–P6 after the sample reached tensile strength) (Figure 14 and Figure 15). In this region a significant difference was found between the strain and the strain rate distribution in the narrow region of the test sample deformation zone. The strain amounts are highest in the central part of the test sample during plastic deformation (points P4–P6), but at the same time, significant strain amounts are found over the entire deformation zone (Figure 14 and Figure 16a). In the same area of the test sample during plastic deformation (points P4–P6), significant localization and the highest amounts of the strain rates are found only in the narrow central region of the deformation zone during plastic deformation (Figure 15 and Figure 16b).

**Figure 17:**
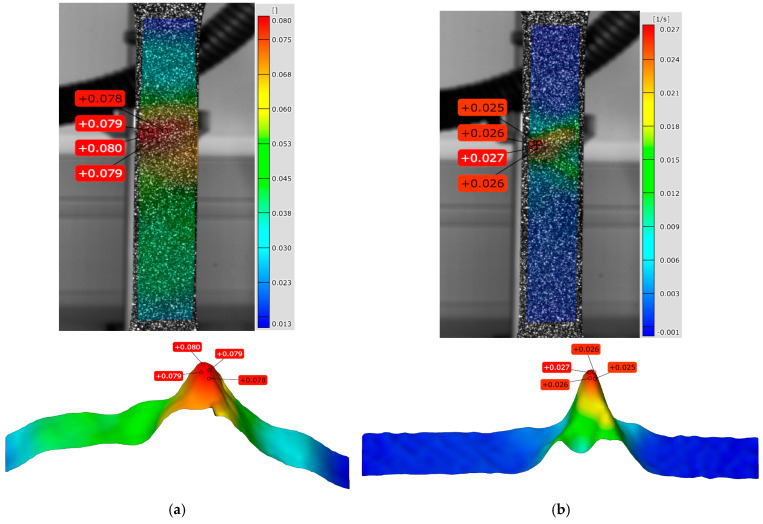
DIC point analysis of (**a**) maximum strain point P6 and (**b**) maximum strain rate point P6.

The maximum strains and strain rates in the testing sample deformation zone were determined (Figure 17). The maximum strain amounts in the central part of the deformation zone were in the range of 0.078–0.080, and the strain rate amounts were in the range of 0.025–0.027 s^−1^ (Figure 17).

The results of the 3D-printed Ti-6Al-4V alloy deformation behavior can be attributed to the fact that as the deformation degree increases, the permanent strain amounts (during plastic deformation) increase over the entire length of the deformation zone. However, the strain rate increases after a certain time only in the narrow central region of the test samples. It is known from the theory of metal forming that in this narrow area, during cold deformation, the most intensive and significant microstructural changes take place during the cold deformation, such as metal hardening with increasing deformation degree. For this reason, a difference between stress, strain, and strain rate distribution over the length of the entire deformation zone was determined, since the changes in the microstructure do not occur only at one specific location but simultaneously in the entire deformation zone due to crystal lattice distortion during cold plastic deformation.

Previous studies have shown that microstructure changes in the 3D-printed Ti-6Al-4V alloy affect its deformation behavior [19]. The processes that occur in the microstructure during cold deformation can be considered as responsible for the deformation behavior of the 3D-printed Ti-6Al-4V alloy. The obtained results of temperature, i.e., stress, strain and strain rate changes indicate that intensive strain hardening processes occur in the microstructure of the 3D-printed Ti-6Al-4V alloy, causing a different stress, strain and strain rate distribution during elastic and plastic deformation. It was determined that these changes are more significant during plastic deformation of the 3D-printed Ti-6Al-4V alloy compared to elastic deformation, as the changes in the microstructure are more significant and intense during cold plastic deformation, where significant hardening of the metal alloys occurs due to an increased deformation degree (Figure 9, Figure 10, Figure 14 and Figure 15).

The importance of the obtained results of stress, strain, and strain rate distribution is demonstrated by the possibility of improving the properties of the 3D-printed Ti-6Al-4V alloy by knowing its deformation behavior. Understanding the stress, strain, and strain rate distribution during metal forming can help in optimizing the printing parameters of the 3D-printed Ti-6Al-4V alloy, such as laser speed, temperature, cooling rate, etc. The stress, strain, and strain rate distribution in the 3D-printed Ti-6Al-4V alloy during the deformation process is important to know since during the 3D printing process the metal is added in layers, where each added layer introduces different stresses during printing. These different residual stresses can later affect the stress, strain, and strain rate distribution during the deformation process. Therefore, the knowledge of stress, strain, and strain rate distribution allows the identification of potentially critical regions of the 3D-printed Ti-6Al-4V alloy during cold deformation. This is because certain areas of the deformation zone of 3D-printed Ti-6Al-4V alloys may be more prone to damage and premature fracture during cold deformation. The reason for the different distribution of local stresses may be the influence of the cooling rate, temperature, and the method of application of the metal layers during the 3D printing process.

In addition to the optimization of parameters in the 3D printing process, the new knowledge gained in this study on the distribution and localization of stresses, strains, and strain rates can be used in the fields of biomedicine (implants and prostheses), the aerospace and automotive industries, and the production of high-precision and sophisticated parts. In biomedical applications, knowledge of the deformation behavior of 3D-printed Ti-6Al-4V alloy is crucial to ensure the functionality, safety, and longevity of biomedical implants and prostheses, given that inhomogeneous distribution of local stresses, strains, and strain rates can lead to damage to implants and prostheses. Therefore, the optimization of stress, strain, and strain rate distribution is extremely important in reducing the risk of excessive local stress, strain and strain rate at specific areas of implants and prostheses. In the aerospace and automotive industries, the safety and reliability of structural components made of 3D-printed Ti-6Al-4V alloy are of significant importance. Therefore, knowledge of its deformation behavior is necessary to ensure a homogeneous distribution of stresses, strains, and strain rates in these parts in order to prevent local concentrations of stresses and strains that can lead to cracks and fractures. Knowledge of the deformation behavior of the 3D-printed Ti-6Al-4V alloy is also crucial for its application in the production of high-precision and sophisticated parts, as even a minor inhomogeneous stress, strain, and strain rate distribution can affect the accuracy and precision of the geometry of components in the field of electronics and optics, where exceptional geometric precision and accuracy of components are required.

However, a completely clear explanation and characterization of the 3D-printed Ti-6Al-4V alloy deformation behavior requires more detailed investigations of the deformation process together with microstructural research. Further research will provide more detailed insight and enable a more precise analysis of 3D-printed Ti-6Al-4V alloy deformation behavior.

## 4. Conclusions

In this research, it is established that it is possible to clearly determine stress, strain, and strain rate distribution using thermography and digital image correlation in 3D-printed Ti-6Al-4V alloy during elastic and plastic deformation.

The following were found through our research:

- The thermoelastic effect in 3D-printed Ti-6Al-4V testing samples during elastic deformation.
- The lowest temperature drop of −0.47 °C for the maximum temperature changes and −0.54 °C for the average temperature changes and the highest temperature amounts of 18.53 °C for the maximum temperature changes and 8.46 °C for the average temperature changes.
- Periodic oscillations of maximum temperature changes during elastic and plastic deformation.
- Different distribution and higher stress, strain, and strain rate amounts during plastic deformation related to the elastic deformation of 3D-printed Ti-6Al-4V alloy.
- Non-uniform strain distribution in the deformation zone of 3D-printed Ti-6Al-4V alloy test samples and both strain and strain rate amounts increasing as the degree of plastic deformation increases.
- The localization of stress, strain, and strain rate in the central region of the 3D-printed Ti-6Al-4V test sample deformation zone.
- The maximum strain amounts are in the range of 0.078–0.080, and strain rate amounts are 0.025–0.027 s^−1^.
- Significant difference between strain and strain rate distribution during localization in the narrow region of the test sample deformation zone during plastic deformation.
- The highest strain amounts in the central region of the test sample and the highest amounts of the strain rates only in the narrow central region of the deformation zone during plastic deformation.

## Figures and Tables

**Table 1:** Parameters of the 3D printing process.

Printing Parameter	Amount
Laser Power	340 W
Laser Speed	1250 mm/s
Layer Thickness	0.06 mm
Hatch Distance	0.12 mm
Energy Input	37.78 J/mm^3^

**Table 2:** Chemical composition of titanium Ti64 Grade 23 powder.

Element, Wt%	Ti	Al	V	O	N	C	H	Fe	Y	Other Elements
Ti64 Grade 23 powder	Balance	5.50−6.50	3.50−4.50	max. 0.13	max. 0.05	max. 0.08	max. 0.012	max. 0.25	max. 0.005	max. 0.40

## Data Availability

The data presented in this study are available in this article.
